# Supplementary material for: Mechanistic basis of the dynamic response of TWIK1 ionic selectivity to pH
Source: Nat Commun. 2024 May 8;15:3849. doi: 10.1038/s41467-024-48067-w (PMC11079055; doi:10.1038/s41467-024-48067-w)
Supplement: Supplementary file 1 — Supplementary Information [file 41467_2024_48067_MOESM1_ESM.pdf]

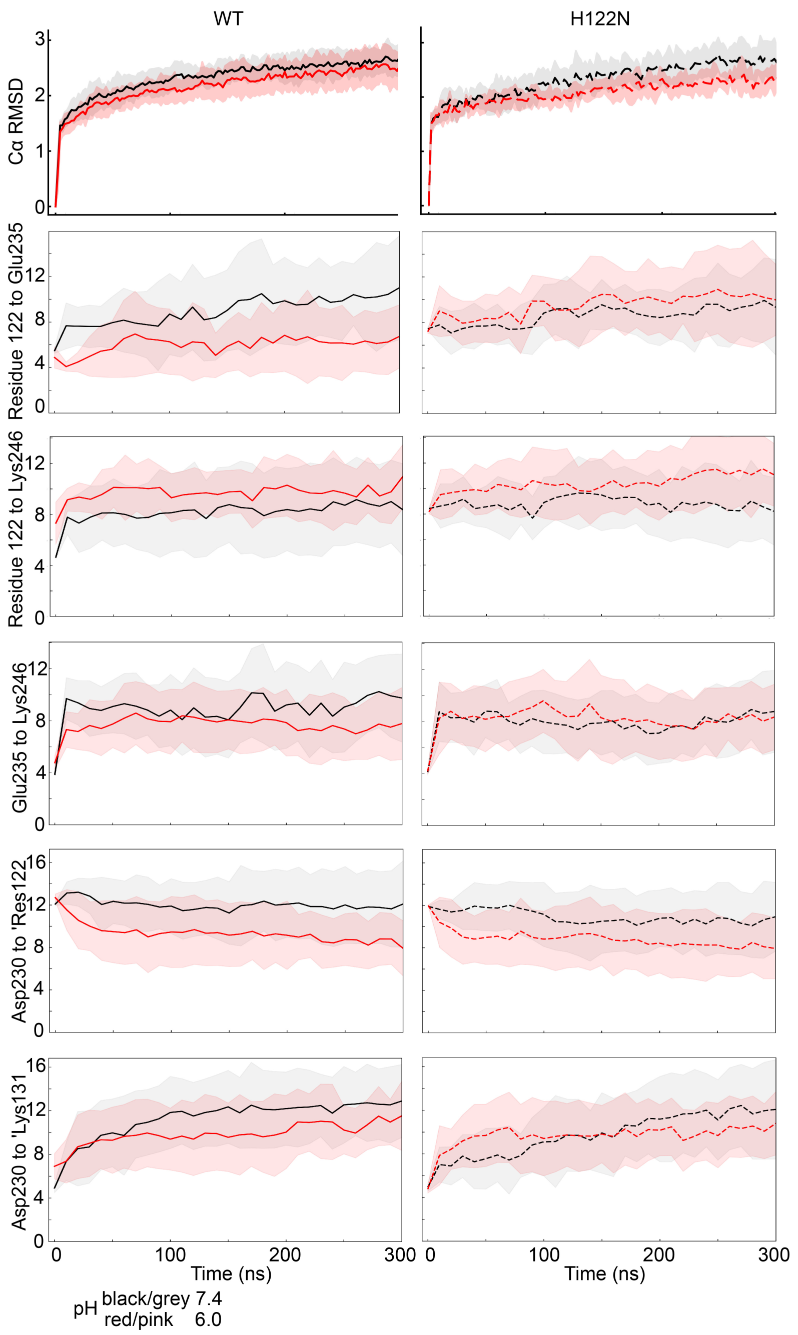

Figure S1. **Time series of the Cα RMSD (top panels) and distances between the centers of mass of side chain heavy atoms of pairs of residues.** Residues are shown on the Y-axis, with distances involving neighbouring subunits identified by the prefix '. RMSD resp. the distances were extracted every 2 resp. 10 ns and compared to the conformation obtained after minimisation and equilibration. At each pH, the RMSD values from 14 resp. 8 individual WT resp. H122N trajectories were extracted, enabling to calculate the mean time evolution of the RMSD and the error (standard deviation). The Lines and dotted lines represent averages, and shaded areas highlight standard deviations. Note that for dissociations of interacting residues (e.g. His122 and Glu235 at pH7.4), convergence is not expected. All values are in Angstroms.

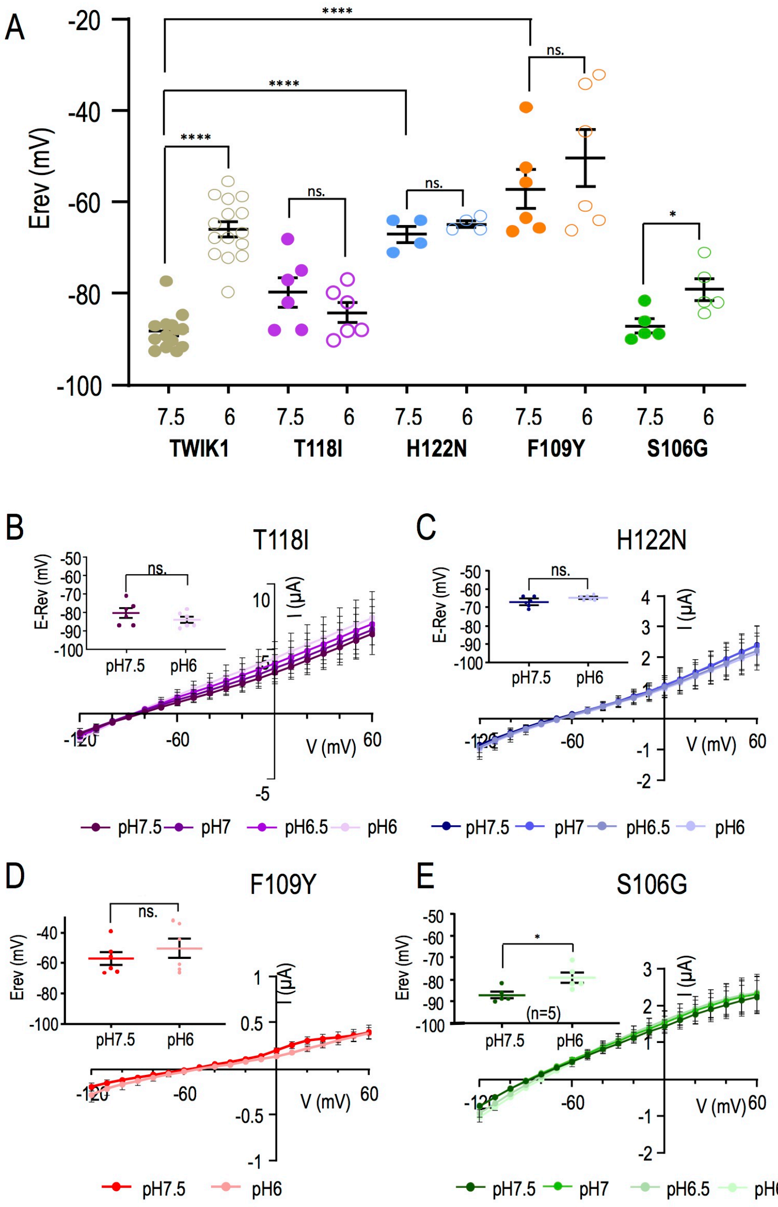

Figure S2. **pH-sensitivity of TWIK1 mutants.** (**A**) Reversal potential (E_{Rev}) variations between pH7.5 (solid dots) and pH6 (empty dots) of TWIK1 (brown), TWIK1.T188I (purple), TWIK1.H122N (blue), TWIK1.F109 (orange) and TWIK1.S106G (green). Values for TWIK1 are from Fig. 1B. Values for TWIK1 mutants T118I, H122N, F109Y and S106G are from Fig. S1. (**B-E**) Currents produced by TWIK1.T118I (**B**), TWIK1.H122N (**C**), TWIK1.F109Y (**D**) and TWIK1.S106G (**E**) in *Xenopus* oocytes at pH7.5, pH7, pH6.5 and pH6. After stabilization of the currents at pH7.5, voltage ramps were applied every ten seconds from -120 mV to +60 mV. pH was changed every 150 sec. Main panels: I/V curves obtained from stabilized currents at different pHs. Inset: reversal potentials (E_{Rev}) measured from stabilized currents at pH7.5 and pH6. All data are presented as mean ± SEM. The number of oocytes examined is 15 (TWIK1), 6 (T118I), 4 (H122N), 6 (F109Y), and 5 (S106G). Statistical relevance has been evaluated using unpaired t-test: ns, non-significant (p=0.27 (A and inset of B), 0.28 (A and inset of C), 0.39 (A and inset of D)); *, p < 0.05 (p=0.024 (A and inset of E); ****, p <0.0001 (A).

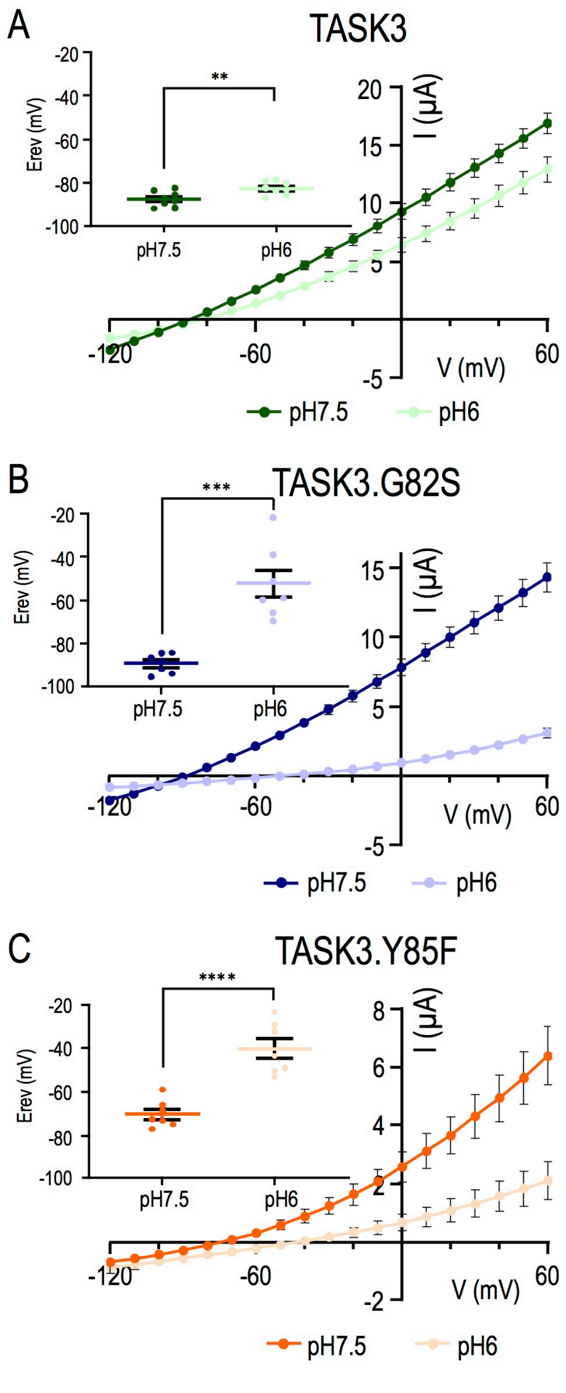

Figure S3. **pH response of currents produced by TASK3 and TASK3 mutants**. (**A-C**) Currents of TASK3 (**A**) and TASK3.G82S (**B**) and TASK3.Y85F (**C**) in *Xenopus* oocytes at pH7.5, Ph7, pH6.5 and pH6. Only currents recorded at pH7.5 and pH6 are shown. The number of oocytes is indicated. Potential ramps were applied from -120 mV to +60 mV. Inset: reversal potentials measured at pH7.5 and pH6. The number of oocytes examined is 8 (TASK3), 7 (TASK3.G82S) and 7 (TASK3.Y85F). Statistical relevance has been evaluated using unpaired t-test: **, p < 0.01 (p=0.008 (A)); ***, p < 0.001 (p=0.0001 (B)). ****, p <0.0001 (C).

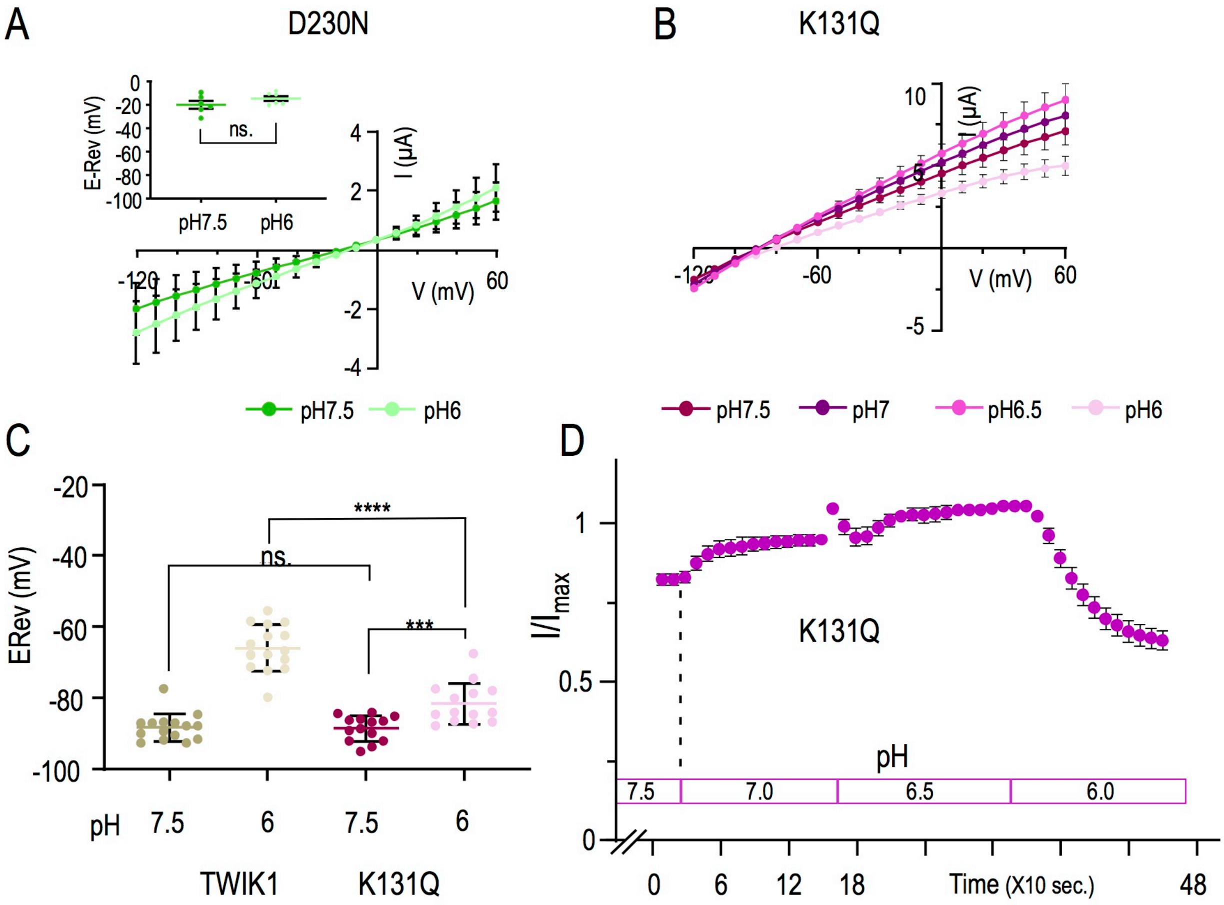

Figure S4. **pH-response of TWIK1.D230N and TWIK1.K131Q currents.** (**A, B**) Currents produced by TWIK1 mutants D230N (**A**) and K131Q (**B**) in *Xenopus* oocytes (the number of oocytes recorded is indicated). After current stabilization at pH7.5, voltage ramps were applied every ten seconds from -120 mV to +60 mV. For K131Q, pH was changed every 150 sec, starting from pH7.5 to pH6. Main panels: I/V curves recorded during the stabilized currents at different pH. Extracted from the voltage ramp recorded at each pH before changing to the next one. Inset: reversal potentials (E_{Rev}) of the stabilized currents at pH7.5 and pH6. (**C**) Reversal potentials (E_{Rev}) of TWIK1 (beige) and TWIK1.K131Q (purple) measured during the stabilized currents at pH7.5 and pH6 (in dark and clear respectively). Values for TWIK1 are from Fig. 1B. (**D**) Kinetics of the TWIK1.K131Q current variations as a function of the pH, measured at 0 mV every 10 seconds. pH changes are indicated. All data are presented as mean ± SEM. The number of oocytes examined is 15 (TWIK1), 6 (TWIK1.D230) and 14 (TWIK1.K131Q). Statistical relevance has been evaluated using unpaired t-test: ns, non-significant ($p=0.20$ (inset of A) and 0.85 (C)); ***, $p < 0.001$ ($p=0.0007$ (C)). ****, $p < 0.0001$ (C).

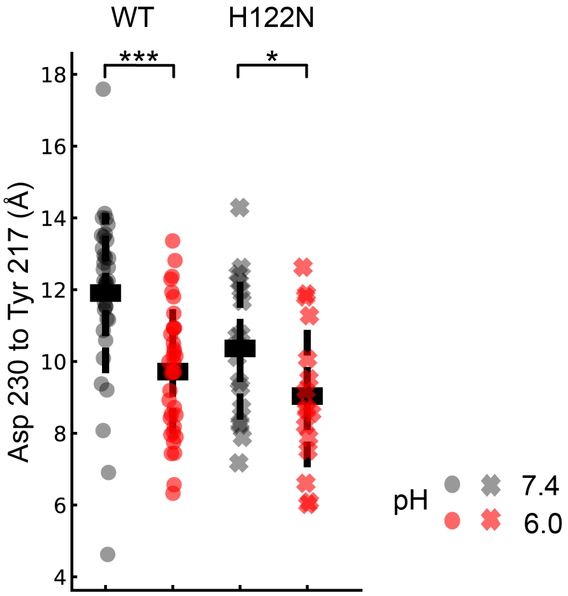

Figure S5. **Distances between Asp230 and Tyr217 at high and low pH.** Distances between the centers of mass of the carboxylic acid functional group and the oxygen atom of the phenolic group. Means (horizontal lines), standard deviation (vertical dashed lines) and the results of an unpaired t-test assuming unequal variance and performed on 18 (WT) resp. 8 (H122N) pairs of values are indicated. The results of statistical analyses are presented as follows: *, $p < 0.05$ (*p = 0.018 (H122N))*; ***, $p<0.001$ (*p=1.00e*$^{-5}$ *(WT)).*

SI-1. Comparison of the CHARMM PBEQ solver with the H++ automated system

As mentioned in the method section, our strategy to estimate the pKas of protein residues involves short MD trajectories (< 10 ns), from which protein conformation, positions of ions interacting with the protein and membrane characteristics (width and width of phospholipid headgroups) are extracted at times 0, 2, 4, 6, and 8 ns. These frames are then subjected to CHARMM's PBEQ module, which determines the total electrostatic potential (PB equation) within 1.5Å grids in a first step, and 0.5Å grids in a second step. For comparison, we subjected the same frames to the H++ automated system, which is faster and less labor-intensive [1]. There is no perfect way of objectively compare these two approaches. Thus, we consider two independent features:

• Residue 122: Histidine 122 has been identified by mutagenesis as a key TWIK1 proton sensor in the range covered by this study [2] (Fig. 1B). It can therefore be used as a reference. Work with CHARMM's PBEQ module yielded a pKa of 6.1 ± 0.7. Fig. S6 shows that the calculated pKa of His122 calculated by H++, using the default internal dielectric constant of 10, was 7.6 ± 2.7. Using internal dielectric constants of 20 resp. 4 resulted in His122 pKa values of 8.7 ± 1.0 resp. 0. Thus, using H++ we would not have been able to validate His122 as a proton sensor.

• Lysine residues: CHARMM's PBEQ identified several lysine residues with a pKa below 6.5, which led us deprotonate them in our work. In our hands using H++, no lysine had a pKa below 10.8. Fig. S7 illustrates a case where Lys131 interacts with the Asp230 from the neighboring subunit. The PBEQ solver provides pKa values of 3.8 for the lysine and 7.3 for the aspartate, suggesting that a proton may be mediating the interaction between these two residues. Using H++, the pKa values are >12 for the lysine and 0.08 for the aspartate. Thus, the module selected for this work seems to better characterize salt bridges and similar interactions. Notably, in a previous computational work studying TWIK1 and in which the pKas were calculated with H++, no lysine residue were identified [3].

These two features support the idea that the CHARMM PBEQ solver is more accurate, justifying its use despite the associated computational costs.

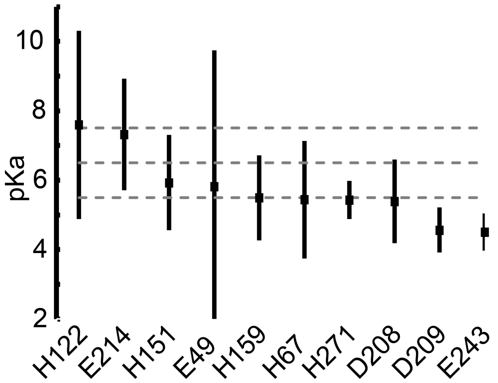

Figure S6. **pKas calculated with H++.** Mean and standard deviations of residues with pKa values close to or within the pH range in which the selectivity of TWIK1 varies are shown. The grey dotted lines indicate this pH range. Frames were extracted at t = 0, 2, 4, 6, and 8 ns of MD simulation. Thus, n = 10, is the number of identical residues in the dimer (2), multiplied by the number of frames extracted from the MD (5).

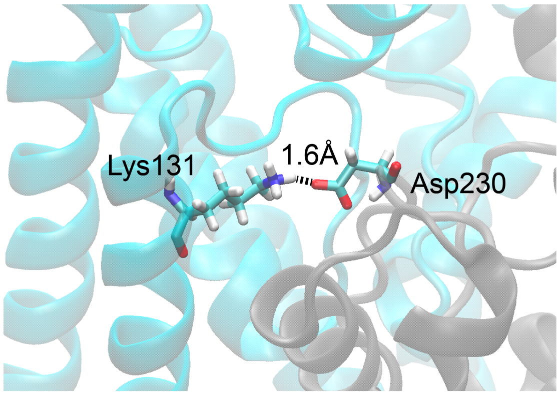

Figure S7. **Proton sharing and pKa calculations using CHARMM and H++.** Molecular representation showing a salt bridge between Lys131 of one subunit (backbone shown in cyan) and Asp230 of another subunit (in grey) during the short MD simulations used for pKa calculations. The distance between the carboxyl oxygen atom of Asp230 and a hydrogen atom of Lys131 is labelled. Only CHARMM pKa calculations suggest proton sharing in this case.

SI-2. Lipid membranes

Biological membranes have a wide variety of lipids and are richer in negative species on the cytoplasmic side. Our approach is based on a compromise between an oversimplified membrane (POPC only) and a "real" one that is difficult to manipulate. Similarly, we ensured that the cytoplasm-facing leaflet was negatively charged. This additional work was carried out only after the pKas had been calculated. To enable the CHARMM PBEQ solver to calculate the pKas of a protein inserted into the membrane, it is possible to specify the thickness of the membrane and the height of the protein relative to the bilayer (insertion level). To our knowledge, it is not possible to specify the lipid composition, but only an overall dielectric constant. In this context, membrane thickness is the main difference between the two membrane systems that is likely to affect the calculations. Thickness influences the overall dielectric constant and, without taking account adaptations in protein structure, a thick membrane will interact with certain residues, whereas these residues may interact with the solvent in the case of a thin membrane. We checked the thickness of the membrane. Fig. S8 shows that the membrane used for pKa calculations (left) and the membrane used for a few randomly selected trajectories (right) are identical. It also shows that membrane thickness neither increases nor decreases over time.

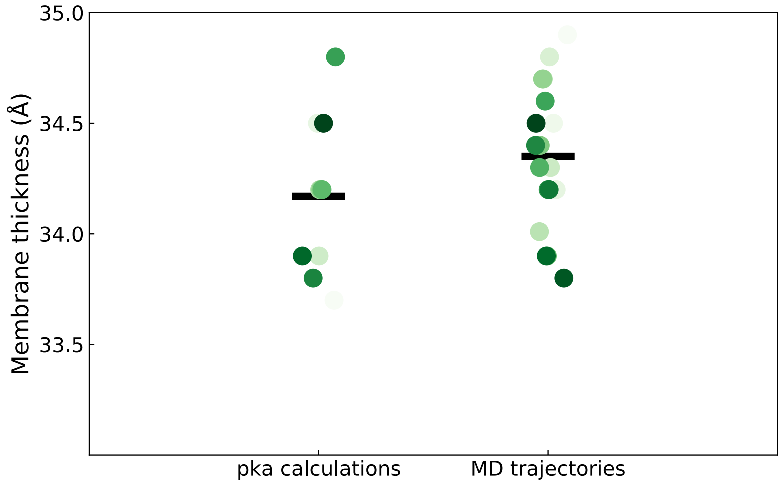

Figure S8**: membrane thickness used for pKa calculations and MD trajectories**. The distances between the phosphorous atoms of the two leaflets are indicated. The color codes, from light to dark green, reflect the values of the images taken at 0-10, resp. 200 ns, where the darkest corresponds to the longest simulation time. n = 10, resp. 18 for values extracted from the pKa calculations resp. MD simulations.

Table S1

pH	Sequence	Protein	Simname	Length (ns)	RMSD	Other analyses
7.4	WT	AB	Repl_01	302	Y	Y
			Repl_02	302	Y	Y
			Repl_03	302	Y	Y
			Repl_04	302	Y	Y
			Repl_05	302	Y	Y
			Repl_06	326	Y	Y
			Repl_07	302	Y	Y
			Repl_08	312	N	Y
			Repl_09	602	N	Y
		CD	Repl_01	302	Y	Y
			Repl_02	302	Y	Y
			Repl_03	302	Y	Y
			Repl_04	302	Y	Y
			Repl_05	302	Y	Y
			Repl_06	326	Y	Y
			Repl_07	302	Y	Y
			Repl_08	312	N	Y
			Repl_09	602	N	Y
6.0	WT	AB	Repl_01	320	Y	Y
			Repl_02	304	Y	Y
			Repl_03	302	Y	Y
			Repl_04	302	Y	Y
			Repl_05	326	Y	Y
			Repl_06	302	Y	Y
			Repl_07	328	Y	Y
			Repl_08	200	N	Y
			Repl_09	200	N	Y
		CD	Repl_01	320	Y	Y
			Repl_02	304	Y	Y
			Repl_03	302	Y	Y
			Repl_04	302	Y	Y
			Repl_05	326	Y	Y
			Repl_06	302	Y	Y
			Repl_07	328	Y	Y
			Repl_08	200	N	Y
			Repl_09	200	N	Y
7.4	H122N	AB	Repl_01	300	Y	Y
			Repl_02	300	Y	Y
			Repl_03	300	Y	Y
			Repl_04	300	Y	Y
		CD	Repl_01	300	Y	Y
			Repl_02	300	Y	Y
			Repl_03	300	Y	Y
			Repl_04	300	Y	Y
6.0	H122N	AB	Repl_01	300	Y	Y
			Repl_02	300	Y	Y
			Repl_03	300	Y	Y
			Repl_04	300	Y	Y
		CD	Repl_01	300	Y	Y
			Repl_02	300	Y	Y
			Repl_03	300	Y	Y
			Repl_04	300	Y	Y

The table shows the protonation state, pH7.4 or pH6.0 as described in the method section, whether TWIK1 or TWIK1.H122N was modelled, the membrane-embedded protein in the double bilayer system ('AB' or 'CD'), the replica, the length of the trajectory, and whether the trajectory was used in the RMSD resp. distance time series shown in Fig. S1. As four TWIK1 pH6.0 trajectories lasted only 200 ns, they were not included in the RMSD calculations.

Supplementary material references

1. Bashford, D. and K. Gerwert, *Electrostatic calculations of the pKa values of ionizable groups in bacteriorhodopsin.* J Mol Biol, 1992. **224**(2): p. 473-86.
2. Chatelain, F.C., et al., *TWIK1, a unique background channel with variable ion selectivity.* Proc Natl Acad Sci U S A, 2012. **109**(14): p. 5499-504.
3. Oakes, V., et al., *Exploring the Dynamics of the TWIK-1 Channel.* Biophys J, 2016. **111**(4): p. 775-784.
