# Supplementary material for: Mechanistic basis of the dynamic response of TWIK1 ionic selectivity to pH
Source: Nat Commun. 2024 May 8;15:3849. doi: 10.1038/s41467-024-48067-w (PMC11079055; doi:10.1038/s41467-024-48067-w)
Supplement: Supplementary file 4 — Source Data [file 41467_2024_48067_MOESM4_ESM.zip › source_data/README_NCOMMS-23-54151.docx]

NCOMMS-23-54151

This README file explains the abbreviations found in the summary_distances_angles and rmsd_values csv tables.

**summary_distances_angles**

Column A: simulated pH value, either 7.4 or 6.0, depending on the protonation states of H122 and D230 (in the region studied in this paper)

Column B: sequence, either WT of H122N

Column C: replicate number.

Column D: length (ns) of the simulations

Column E: Since two channels (dimers) were investigated in each simulation, there are a total of four different subunits in each box. Subunits a and b refer to one channel, and subunits c and d refer to the other channel.

Columns F to L:

Res_122_sc_GLU235_OE_within: distances between the COM of the residue 122 sidechain and the COM of the carboxylic acid functional group of E235 of the same subunit.

Res_122_sc_LYS246_NZ_within: distances between the COM of the residue 122 sidechain and the K246 sidechain terminal nitrogen atom of the same subunit.

GLU235_OE_LYS246_NZ_within: distances between the COM of the carboxylic acid functional group of E235 and the K246 sidechain terminal nitrogen atom of the same subunit.

PHE109_ring_LYS246_NZ_within: distances between the COM of the benzoyl functional group of F109 and the K246 sidechain terminal nitrogen atom of the same subunit.

ASP230_carb_H122_ND1_NE2_inter: distances between the COM of the carboxylic acid functional group of D230 and the COM of the residue 122 sidechain of the neighboring subunit.

Res_122_sc_SER86_OG_within: distance between the COM of the residue 122 sidechain and the alcoholic functional group of S86 of the same subunit.

ASP230_carb_LYS131_NZ_inter: distances between the COM of the carboxylic acid functional group of D230 and the K246 sidechain terminal nitrogen atom of the neighboring subunit.

Remarks:

1. Residue 122 sidechain: For His, the sidechain heavy atoms are the two nitrogen atoms (ND1, NE2), whereas for Asn, the carboxamide functional group (OD1 and ND2) was used.
2. In all cases, heavy atoms only were considered.

Column M:

Orientation of residue 122 as described in the main article. To build the tip of the sidechain, the same atoms were used as for the distance calculations.

**rmsd_values**

Column A: simulated pH value, either 7.4 or 6.0

Column B: sequence, either WT of H122N

Column C: replicate number.

Column D: Two channels were investigated in each simulation, either channel ‘AB’ or ‘CD’

Columns E to FM:

RMSDs of the Ca atoms, reported at 2 ns intervals and in Angstrom.
